# Unlocking the chemistry of bile acids for cancer therapeutics

**DOI:** 10.1186/1755-8166-7-S1-I48

**Published:** 2014-01-21

**Authors:** Avinash Bajaj

**Affiliations:** 1 Laboratory of Nanotechnology and Chemical Biology, Regional Centre for Biotechnology, Gurgaon, India

## 

Breast cancer is the second leading cause of cancer deaths today after lung cancer and is the most common cancer among women. The primary drug tamoxifen is used treat breast cancer has several problems including poor oral bioavailability. Bile acids/salts are known to be components of endogenous molecular pool that solubilizes, absorbs dietary fat/lipid molecules in the form of micelles. Bile acids are interesting chemical scaffold for drug conjugation due to presence of different number of free hydroxyl groups and a free carboxylic acid. We have been exploring bile acids as drug carriers for cancer therapy. We used three bile acids: lithocholic acid (LCA), deoxycholic acid (DCA) and cholic acid (CA) to engineer bile acid tamoxifen conjugates with free amine and acid functionalities. In this talk, I would present the interactions of these new drug carriers and their therapeutic potential for breast cancer therapy.

